# Base-By-Base version 2: single nucleotide-level analysis of whole viral genome alignments

**DOI:** 10.1186/2042-5783-1-2

**Published:** 2011-06-14

**Authors:** William Hillary, Song-Han Lin, Chris Upton

**Affiliations:** 1 Biochemistry and Microbiology, University of Victoria, 213 Petch Building, Ring Road, Victoria, B.C., V8W 3P6, Canada

## Abstract

**Background:**

Base-By-Base is a Java-based multiple sequence alignment editor. It is capable of working with protein and DNA molecules, but many of its unique features relate to the manipulation of the genomes of large DNA viruses such as poxviruses, herpesviruses, baculoviruses and asfarviruses (1-400 kb). The tool was built to serve as a platform for comparative genomics at the level of individual nucleotides.

**Results:**

In version 2, BBB-v2, of Base-By-Base we have added a series of new features aimed at providing the bench virologist with a better platform to view, annotate and analyze these complex genomes. Although a poxvirus genome, for example, may be less than 200 kb, it probably encodes close to 200 proteins using multiple classes of promoters with frequent overlapping of promoters and coding sequences and even some overlapping of genes. The new features allow users to 1) add primer annotations or other data sets in batch mode, 2) export differences between sequences to other genome browsers, 3) compare multiple genomes at a single nucleotide level of detail, 4) create new alignments from subsets/subsequences of a very large master alignment and 5) allow display of summaries of deep RNA sequencing data sets on a genome sequence.

**Conclusion:**

BBB-v2 significantly improves the ability of virologists to work with genome sequences and provides a platform with which they can use a multiple sequence alignment as the basis for their own editable documents. Also, a .bbb document, with a variety of annotations in addition to the basic coding regions, can be shared among collaborators or made available to an entire research community. The program is available via Virology.ca using Java Web Start and is platform independent; the Java 1.5 virtual machine is required.

## Background

The original version of Base-By-Base (BBB) [1] was developed by the Virus Bioinformatics Resource Center primarily because of a need for a customizable, platform-independent (Java), multiple sequence alignment (MSA) editor that could be used for the comparison, annotation and analysis of viral genomes, and also integrated directly with our MySQL database. There are now several somewhat related MSA tools; however, each has their own set of distinctive features that make them especially valuable in their niche environments. For example, JalView [2] and STRAP [3] focus on features for display and analysis of proteins, whereas SeaView [4] adds phylogenetic analyses to a nucleic acid alignment editor. Key distinctive features of the original BBB were its ability to read annotations (CDS positions) from GenBank files, an explicit display of differences between sequences and a tool to compare genomes, including the ability to summarize the consequences of all nucleotide changes on encoded proteins throughout a genome. These basic features have been retained and frequently improved upon in Base-By-Base version 2 (BBB-v2), and a series of new features have been added.

Throughout the development of BBB-v2, our focus has been on the user experience. We have endeavoured to make the tool intuitive and to add features that are useful to molecular biologists working daily with large viral genomes (up to 300 kb) that contain approximately 1 gene/kb of sequence. To this end, we also link BBB-v2 to our graphical database interface so that it can be used to select genomes, genes and proteins, which are then opened in BBB; this provides much easier access to data.

## Results and Discussion

Many of the features of version 1 of BBB were intended to optimize the visualization of MSAs; for example, using a novel display of differences between sequences, collection and summary of all differences between pairs of sequences, providing a summary view on complete MSAs and the display of the results of motif searches through the sequences. In BBB-v2, the addition of a variety of new features creates a more interactive workspace/notebook for the bench scientist.

### User annotation of sequences

**Figure 1:**
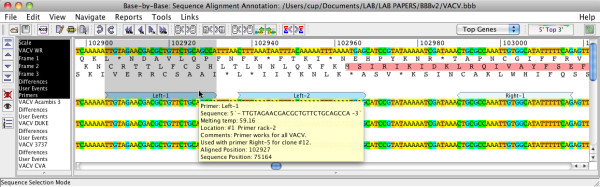
**Annotation of VACV-WR with primer sequences**. A section of the VACV-WR genome, with 3-frame translation, is displayed at the top of the MSA together with 3 primer annotations. ***Mouse-over ***of the primer region shows primer information, which is also searchable from within BBB-v2. Although in this view, BBB-v2 shows the ***top strand ***of the genome with the rightward-transcribed gene highlighted in pink, primers associated with both DNA strands are always shown; primers associated with the non-selected strand are shown in a paler colour.

Navigation through complex MSAs of many large sequences is usually very cumbersome and notes are normally kept separate from the alignment in a notebook or on a multi-sheet paper copy of the alignment. BBB allowed users to add comments to any sequence in a MSA, which were displayed in the MSA window and could also be used for navigation through the alignment. At the request of a user, in BBB-v2 we have used a similar style of annotation, for interface consistency, to associate DNA primer sequences with an alignment sequence (see menu: **Tools/Primer**). The primer sequences can be added singly or in a batch from a text file in which *primer sequence *and *primer name *are essential data items whereas *freezer locations, Tm *and *comments *represent optional information (Figure 1). The primer sequences, which need not be perfect matches to the DNA sequence, are found within the MSA sequences using a fuzzy-sequence matching algorithm, and the information is added to the .bbb XML file, which must be save locally. The primer information can be navigated and searched in the same way that comments are searched; it could serve as a resource for an entire lab.

**Figure 2:**
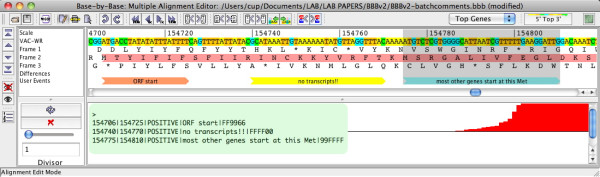
**Addition of multiple comments using a text file**. A section of the VACV-WR genome, with 3-frame translation, is displayed with RNA sequencing data (see below). The comments were added by using the File/Import Analysis from File menu with the test illustrated in the green box. Most orthologs of this thymidylate kinase gene are annotated to start at the 2**^nd ^**methionine of this VACV-WR ORF; the RNA sequencing data displayed in the lower panel supports a similar conclusion for VACV-WR.

A second new annotation method allows the temporary *decorating *of a sequence or multiple sequences within a MSA with data from an external analysis, for example the prediction of promoter motifs in a viral genome (see menu: **File/Import Analysis from File)**. Simply, a series of *Comment *arrows are drawn connected to a sequence, in the MSA window, following the instructions in a text file that specify *start/stop *position for the arrow, *strand *of DNA (POSITIVE/NEGATIVE) with which to associate the comment, *description *(text) and *color *(hexadecimal format). Figure 2 shows the use of a manually edited text file to display 3 comments with information about the start of the VACV-WR thymidylate kinase gene with RNA sequencing data that suggests the correct gene start is the 2^nd ^methionine of the open reading frame.

Finally, we became aware that it would be very useful to attach a variety of information to particular MSAs that were being used for multiple analyses. For example, the name of the program used for alignment with any deviations from default settings, manual edits made to the MSA and results of previous searches and analyses. To this end, we added a new module to BBB-v2 to allow textual information (up to 500 lines) to be incorporated into the .bbb file. This is edited and saved together with the MSA as a single file, so there is no extra work involved or chance that important notes will be lost (see menu: **Edit/Edit MSA Notes)**.

### Manipulation of sequences within a MSA

After aligning and viewing sequences/genomes retrieved from web-based resources, the researcher may still face a number of problems. These large sets of sequences often contain multiple copies of identical sequences from different virus isolates. BBB-v2 offers the ability to remove these extra sequences (see menu: **Edit/Remove identical sequence(s)**). Likewise, sequences from these resources often have complex names with Accession, Locus and Version information that obscures viewing of more human-intelligible names. To simplify the process of changing names, BBB-v2 has an easy to use tool to edit the sequence names (see menu: **Edit/Edit Sequence Names**).

Furthermore, the viewing and maneuvering through very large MSAs is often slow and usually tedious. One possibility to deal with this problem may be to save a copy of the alignment and remove some sequences (see menu: **File/Remove Sequences**) from the MSA, however, this doesn't overcome problems with long genomic sequences. Therefore in BBB-v2 we have incorporated several simple methods to create new alignments from subsets of the sequences and also to use sub-sequences. After selecting the required subset of sequences in the MSA, the researcher can also select a region of the sequences before 1) Saving to a .bbb file (see menu: **File/Save Selected Regions to BBB file**), 2) opening in a new alignment window (see menu: **File/Open Selected Regions to new BBB file**), or 3) Sending to a new text file (see menu: **File/Export Selected Regions to fasta**). These features allow users to maintain a *master *MSA for reference purposes, but to work on different subsets of the data when expeditious; user annotations are also exported with the subsequences.

### Display of sequence variability within a MSA

**Figure 3:**
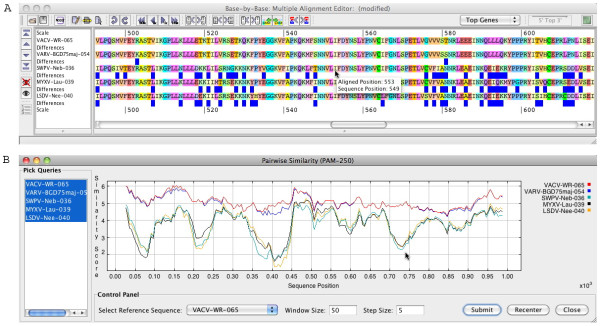
**Comparison of sequences**. a) Alignment of 5 poxvirus DNA polymerase proteins. In this example, the ***blue boxes***, shown under a sequence, indicate a difference to the top sequence in the MSA. b) Similarity plot calculated with a PAM250 matrix of the MSA shown in (a). The ***Reference Sequence***, ***Window Size ***and ***Step Size ***can be changed, and the user can zoom into a particular region by using the cursor to draw box on the plot; ***Re-centre ***returns the plot to the full size.

Although the display of aa/nt differences between adjacent sequences was a feature of BBB and provides a very visual representation of sequence variation, including a *Visual Summary *(see menu: **Reports/Visual Summary**), users requested additional flexibility in this display. Therefore we have implemented two additional methods for showing differences within a MSA: *compare against consensus *(see menu: **View/Comparison Method/Against Consensus**) and *compare to the top sequence in alignment *(Figure 3a) (see menu: **View/Comparison Method/Against Top Sequence**). The latter allows the researcher to choose any sequence as the reference since BBB-v2 allows the user to easily re-order the sequences within the MSA.

If the length of the sequences and/or the number of differences between them is large, the *Visual Summary *display may be inadequate for resolving single events; therefore the differences can be exported from a BBB-v2 alignment to a text file suitable to importing into our genome browser, the Viral Genome Organizer [5] (see menu: **Tools/Export Differences to VGO**). A user may require this feature to get an overall picture of the distribution of differences along two genomes being compared.

Alternatively, a new feature that displays identity plots for DNA sequences and similarity plots for protein sequences (PAM250 matrix) can be used to show the relationship between multiple sequences in an alignment (see menu: **Reports/Sequence Similarity Graph**). This feature was inspired by SimPlot [6] and allows the user to change the size of the *Sliding Window *and the *Step Size *as well as to choose which sequence from the MSA should be used as the reference (Figure 3b). A user can also *Zoom *into a region of the plot.

One of the unique features of BBB was the ability to use annotations from a sequence to identify differences in genes within 2 genomes being compared; this was intended for assessing variation between very similar genomes. BBB-v2 now can summarize the differences among multiple genomes and was used in a comparison of varicella zoster genomes from vaccinated individuals [7,8].

### Display of deep RNA sequencing data

**Figure 4:**
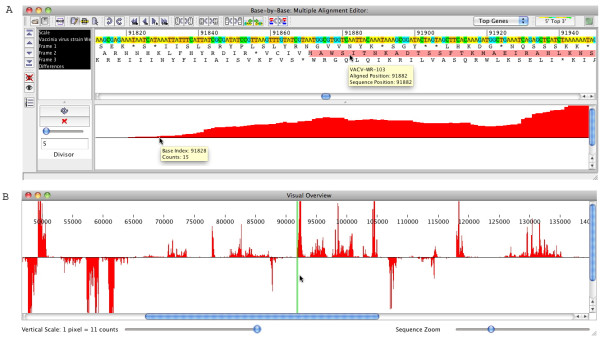
**Mapping of RNA sequencing data to VACV-WR genome in BBB-v2**. (a) Upper panel shows genome sequence and 3-frame translation. BBB-v2 displays position of gene WR-103 (VLTF-4); gene name is displayed by ***mouse-over***. Lower panel plots the MochiView data (RNA sequence counts) at each nucleotide position. The scale of the plot can be changed using the slider at the left or typing a value into the ***Divisor ***box; in this plot, 1 pixel in height represents a count of 5 sequences. The actual count value and nucleotide position is displayed by ***mouse-over ***above the plot. (b) The ***Visual Overview ***allows the user to get a birds-eye view of the RNA sequence counts over the complete genome. The vertical scale can be changed with the slider on the left and the ***Sequence Zoom ***slider allows the user to magnify the horizontal scale approximately 60 fold. The vertical green lines (updated live) encompass the region of the sequence that is currently displayed in the primary BBB-v2 window.

During the design of BBB-v2, we have focused on adding features that help the bench scientist manipulate and view information present in multiple alignments. However, there are other types of data that need to be displayed at a nucleotide level, but across relatively large sequences. An example is the data from transcriptome mapping by deep RNA sequencing. Although there are multiple software tools to work with Next Generation Sequence data, these tend to be too complex for the casual user who wants to browse the "bottom line" results, which can be summarized as "number of sequences mapping to a particular nucleotide". Recently, Yang *et al*. published the mapping of early VACV-strain WR transcripts using deep RNA sequencing [9] and kindly supplied us with a MochiView [10] summary file that lists every base-pair position in the VACV-strain WR genome together with the number of sequencing reads that map to it (both plus and minus sense). While attempting to view the data in MochiView, it became apparent that a simpler system would be necessary for most virologists to be able to make use of this wonderful data set and the many more to come as researchers begin to use Next Generation Sequencing tools. Therefore we added a new module to BBB-v2 to plot this type of transcriptome data in association with a genome sequence and its gene annotation information. This transcription data is available in two complementary views (Figure 4; Additional files 1 and 2). The first is as a plot directly below the sequence (x-axis) with a scalable *number of RNA sequences *(y-axis) to allow viewing of the complete data range. This is similar to the way a bar graph is used to represent conservation in the consensus plot of BBB. The second provides a view in a new window and can be zoomed-out along the x-axis to display the entire poxvirus genome as well as changing the scale of the y-axis. Although Next Generation Sequencing data, by necessity, is processed a number of times before it reaches the user, it is important to keep the researcher as close as possible to the raw data. Since BBB was originally created to support bioinformatics of poxviruses (and other viruses), this new display feature should allow virologists to visualize most of the key information from such deep RNA sequencing studies without needing to learn a new and complex software package. Other types of data could also be plotted if the input was formatted similarly.

## Conclusions

A significant number of new features have been added to the original BBB alignment editor. Although at the heart of this program is the ability to display and edit MSAs, it is unique in that many of its functions focus on the analysis and annotation of viral genomes, sequences in the 10-300 kb range. BBB-v2 also aims to present the user, a bench scientist, with a MSA that can be further analyzed/searched and annotated by the user. In BBB-v2, we have added 2 new categories of annotation in addition to the *sequence comments *that could be attached to these regions of sequences in BBB. The ability to save genome-linked primer sequences with a variety of annotations, together with an editable text page allows the research scientist to use a particular MSA .bbb document as an extension of their lab notebook.

The addition of a module to view MochiView summary data provides virologists a familiar intuitive way to examine RNA sequencing data that is critical for development of new, more accurate transcription maps of viral genomes.

## Methods

Essentially, BBB-v2 builds directly upon the original BBB Java code, which was chosen to enable support of Mac OS X, MS Windows and LINUX computer platforms. A user opens the application (client) using Java Web Start from a Virology.ca web page. This approach, which automatically downloads the application from the host server computer whenever a new version is available, greatly simplifies the distribution of updates and ensures users are taking advantage of the latest version of the software.

BBB was developed to be used as an editor for MSAs generated from the Virus Orthologous Clusters (VOCs) [11] database at Virology.ca [12], with sequences being selected in the VOCs GUI, sent to an alignment program (ClustalW [13], T-Coffee [14] or MUSCLE [15]) and finally displayed in BBB. However, the program can also save/load alignments and sequences from the user's local computer; these can be in FASTA, GenBank (read only) or the native file format .bbb, which is based on the XML Bioinformatics Sequence Markup Language (BSM) standard. The BBB file format is required if the user needs to store information other that the sequence alignment, such as CDS features, DNA primers and user-added annotations for the sequences. The XML format of BBB files also simplifies the sharing of this information between tools.

Relatively small alignments can be performed within BBB, but because users may have favourite tools or specific needs in the alignment of large DNA sequences it is expected that large alignments from tools such as DIALIGN [16] or MAFFT [17] will be loaded as FASTA alignments (output files of these programs). Importantly, BBB-v2 permits the user to add genome annotations, for example from a GenBank file, back to a gapped sequence within a MSA after alignment of FASTA formatted sequences in another program.

## Availability and requirements

**Project name: **Base-By-Base

**Project home page: **http://athena.bioc.uvic.ca/tools/BaseByBase

**Operating system(s): **Platform independent

**Programming language: **Java

**Other requirements: **Java 1.5 or higher, this requires at least system 10.5 on Apple OS X.

**License: **GNU General Public License

**Any restrictions to use by non-academics: **Contact authors

## Competing interests

The authors declare that they have no competing interests.

## Authors' contributions

WH and SL were principle programmers of the BBB-v2 software. CU contributed ideas for features and display requirements, tested the program and wrote the manuscript. All authors have read and approved the manuscript.

## Supplementary Material

Additional file 1 **VACV-WR**. This file contains the VACV strain Western Reserve genome in GenBank format, including gene annotations. Click here for file

Additional file 2 **mochi-wr-example**This file contains MochiView summary data for RNA sequencing from a sample isolated 1 hr after infection by VACV-WR. It should be loaded into BBB-v2 with the VACV-WR.gb file. Click here for file
